# The Impact of Childhood Maltreatment on Emotional Dysregulation in Adults with ADHD: A Comprehensive Study

**DOI:** 10.1192/j.eurpsy.2025.739

**Published:** 2025-08-26

**Authors:** G. Avci Buyukdogan, S. Sertcelik, M. Nebioglu Yildiz

**Affiliations:** 1 Psychiatry Clinic, Health Sciences University Istanbul Sultan Abdulhamid Han Research and Training Hospital; 2 Psychiatry Clinic, Health Sciences University Istanbul Haydarpasa Numune Research and Training Hospital, Istanbul; 3 Psychiatry Clinic, Mersin University, Mersin, Türkiye

## Abstract

**Introduction:**

Attention Deficit/Hyperactivity Disorder (ADHD) is a neurodevelopmental disorder characterized by symptoms of inattention, hyperactivity, and impulsivity, which adversely affect an individual’s functioning across various domains, including home, work, and social environments. Recent research has underscored the presence of emotional dysregulation in individuals with ADHD, contributing to significant clinical and functional impairments.

**Objectives:**

This study aims to investigate the presence and severity of emotional dysregulation and childhood maltreatment in adults with ADHD and to explore the potential association between emotional dysregulation and childhood maltreatment within this population.

**Methods:**

The study included 80 newly diagnosed adult patients with ADHD and 80 healthy control participants. ADHD diagnosis or exclusion was conducted using the Diagnostic Interview for ADHD in Adults (DIVA 2.0) based on DSM-5 criteria. Additionally, the ADHD group underwent screening with the DSM-IV Structured Clinical Interview (SCID-I). Participants were assessed using the Difficulties in Emotion Regulation Scale (DERS), the Childhood Trauma Questionnaire (CTQ), the Beck Depression Inventory (BDI), the Beck Anxiety Inventory (BAI), and ADHD self-report scales (WURS and ASRS).

**Results:**

The ADHD group exhibited significantly higher total and subscale scores on the DERS compared to the healthy control group (p<0.001). Similarly, except for the “physical neglect” subscale, all subscale scores and the total score on the CTQ were significantly elevated in the ADHD group relative to the control group (p<0.001). Significant correlations were identified between the subscales and total scores of the DERS and CTQ. Depression and anxiety levels emerged as predictors of emotion regulation difficulties within the ADHD group. Across the entire sample, childhood trauma exposure (CTQ score), ADHD symptoms in adulthood (ASRS score), and levels of depression and anxiety were identified as predictors of emotion regulation difficulties.

**Conclusions:**

The findings indicate that adults with ADHD exhibit more pronounced emotional dysregulation and higher levels of childhood maltreatment compared to healthy controls. Furthermore, childhood maltreatment appears to exacerbate emotional dysregulation in this population.

**Disclosure of Interest:**

None Declared

